# Purified Mesenchymal Stem Cells Are an Efficient Source for iPS Cell Induction

**DOI:** 10.1371/journal.pone.0017610

**Published:** 2011-03-11

**Authors:** Kunimichi Niibe, Yoshimi Kawamura, Daisuke Araki, Satoru Morikawa, Kyoko Miura, Sadafumi Suzuki, Shigeto Shimmura, Takehiko Sunabori, Yo Mabuchi, Yasuo Nagai, Taneaki Nakagawa, Hideyuki Okano, Yumi Matsuzaki

**Affiliations:** 1 Department of Physiology, Keio University School of Medicine, Tokyo, Japan; 2 Department of Dentistry and Oral Surgery, Keio University School of Medicine, Tokyo, Japan; 3 Department of Ophthalmology, Keio University School of Medicine, Tokyo, Japan; Instituto Nacional de Câncer, Brazil

## Abstract

**Background:**

Induced pluripotent stem (iPS) cells are generated from mouse and human somatic cells by the forced expression of defined transcription factors. Although most somatic cells are capable of acquiring pluripotency with minimal gene transduction, the poor efficiency of cell reprogramming and the uneven quality of iPS cells are still important problems. In particular, the choice of cell type most suitable for inducing high-quality iPS cells remains unclear.

**Methodology/Principal Findings:**

Here, we generated iPS cells from PDGFRα^+^ Sca-1^+^ (PαS) adult mouse mesenchymal stem cells (MSCs) and PDGFRα^−^ Sca-1^−^ osteo-progenitors (OP cells), and compared the induction efficiency and quality of individual iPS clones. MSCs had a higher reprogramming efficiency compared with OP cells and Tail Tip Fibroblasts (TTFs). The iPS cells induced from MSCs by Oct3/4, Sox2, and Klf4 appeared to be the closest equivalent to ES cells by DNA microarray gene profile and germline-transmission efficiency.

**Conclusions/Significance:**

Our findings suggest that a purified source of undifferentiated cells from adult tissue can produce high-quality iPS cells. In this context, prospectively enriched MSCs are a promising candidate for the efficient generation of high-quality iPS cells.

## Introduction

Pioneering work by Takahashi et al showed that the ectopic expression of a defined set of transcription factors, Oct4, Klf4, Sox2, and c-Myc, reprograms mouse embryonic fibroblasts (MEFs) and adult tail-tip fibroblasts (TTFs) into embryonic stem (ES)-like cells called induced pluripotent stem (iPS) cells [1]. Since then, iPS cells have been generated from a variety of somatic cells, including embryonic and adult dermal fibroblasts [1], [2], [3], epithelial cells of the liver and stomach [4], pancreatic β cells [5], mature B lymphocytes [6], and adult neural stem cells (NSCs) [7], [8]. These studies demonstrated that most somatic cells can be reprogrammed with 4 or 3 factors (excluding c-Myc).

However, each cell source may have a unique requirement for the specific factors that induce reprogramming. For example, embryonic fibroblasts are more easily reprogrammed than adult ones [1], [9]. Mature B cells require an additional factor to trigger epigenetic change, whereas NSCs require only 1 or 2 factors to become iPS cells. These data raise two possibilities: 1) embryonic tissue is a better source for iPS cells than adult tissue, and 2) tissue stem cells are more suitable for reprogramming than differentiated cells. However, it is difficult to compare the reprogramming efficiency among mixed cell populations such as MEFs or TTFs. Furthermore, nothing conclusive can be learned from comparing cells of different lineages, such as B lymphocytes versus NSCs. Somatic cells constitute a developmental hierarchy of stem cells, progenitor cells, and mature cells. To test our hypothesis that stem cells are more efficiently reprogrammed into iPS cells than mature ones, we needed to compare cells from the same cell lineage but from distinct developmental stages.

**Figure 1:**
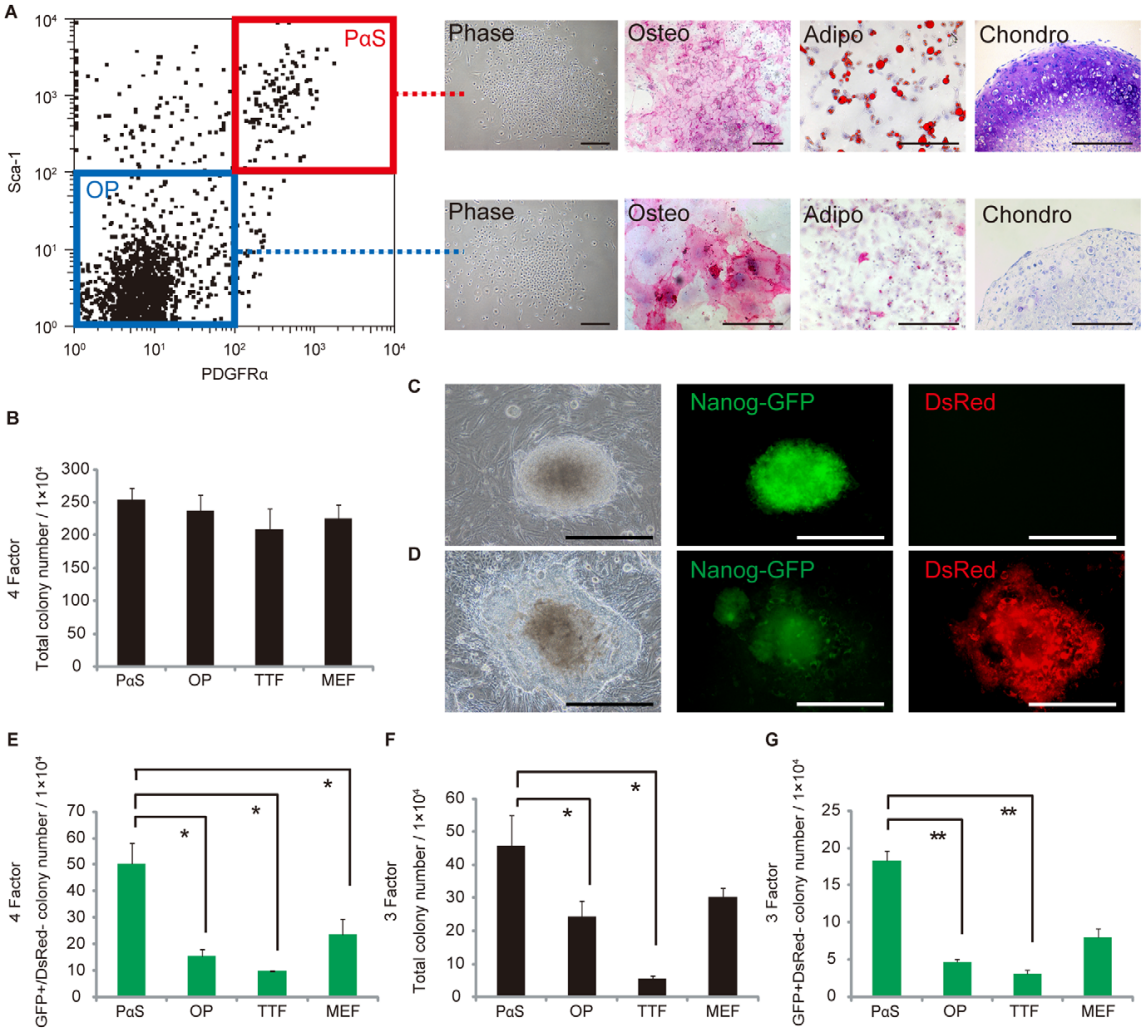
Generation of iPS cells from three cell sources obtained from Nanog-GFP-Puro^r^ transgenic mice. **A**, FACS profile and differentiation capacity of PαS cells and OP cells. PαS cells positive for PDGFRα and Sca-1 after gating on CD45^−^ and Ter119^−^. OP cells negative for PDGFRα and Sca-1 after gating on CD45^−^ and Ter119^−^. Phase-contrast is a micrograph of CFU-Fs from PαS cells. Osteogenesis was indicated by alkaline phosphatase staining on day 14, adipogenesis by neutral lipid vacuoles stained with oil red O on day 14, and chondrogenesis by toluidine blue staining on day 21 and by morphological changes of PαS (top) and OP (bottom) cells. **B**, Number of total colonies (counted EGFP^+^ and EGFP^−^ cells) obtained from 4Factor-transfection of PαS cells, OP cells, TTFs, and MEFs. Colonies were counted 28 days after retroviral transduction and 1×10^4^ cells were replated. **C**, Phase and fluorescence images of PαS iPS GFP^+^/DsRed^−^ colony. **D**, Phase and fluorescence images of PαS iPS GFP^+^/DsRed^+^ colony. **E**, Number of GFP^+^/DsRed^−^ colonies from 4-factor induction. *P<0.05 (n = 3). **F**, Number of total colonies (counted EGFP^+^ and EGFP^−^ cells) from 3-factor induction. Colonies were counted 35 days after retroviral transduction and 1×10^4^ cells were replated. *P<0.05 (n = 3). **G**, Number of GFP^+^/DsRed^−^ colonies from 3-factor induction. **P<0.01 (n = 3). Bar, 200 µm.

Here we focused on highly enriched mesenchymal stem cells (MSCs) and osteo-progenitors. Both cell types belong to the mesenchymal lineage and maintain unique undifferentiated states. We previously established a method for isolating highly enriched MSCs and osteo-progenitors from adult murine bone marrow based on their expression of PDGFRα and Sca-1. Cells expressing both PDGFRα and Sca-1 (PαS) are MSCs that are 120,000-fold more enriched for clonogenic cells (CFU-Fs [10], [11], [12]) than unfractionated bone marrow [13], [14]. On the other hand, cells in the PDGFRα-negative, Sca-1-negative represents osteo-progenitors (OP) that can differentiate only into osteocytes (**Figure 1A**). We isolated each population to compare the efficiency of reprogramming.

## Results

### Generation of iPS cells with distinct subsets of mesenchymal lineage

Each isolated cell type (PαS, OP, Tail Tip Fibroblast (TTF), and Mouse Embryonic Fibroblast (MEF)) was retrovirally transduced with 4 or 3 factors [15] along with CAG-DsRed [16] as a control for the induction efficiency, which was similar for both cell populations (**Figure S1**). From 1×10^4^ DsRed-positive cells induced with 4 factors, we obtained over 200 iPS colonies from the PαS and OP cells, which was approximately the same amount obtained with control TTFs and MEFs counted 35 days after retroviral transduction (**Figure 1B**). Nanog is specifically expressed in ES cells and pre-implantation embryos [17], [18] and is an indicator for pluripotency during iPS-cell induction [19]. The Nanog GFP^+^/DsRed^−^ colonies were morphologically indistinguishable from mouse ES cells (**Figure 1C**), but the GFP^+^/DsRed^+^ colonies showed slightly flat with unclear margins (**Figure 1D**). From what we found in the comparison of GFP^+^/DsRed^−^ colonies was that the PαS cell-derived iPS cells (PαS-iPS) included significantly more GFP^+^/DsRed^−^ and puromycin-resistant colonies than the OP-, TTF-, and MEF-derived iPS cells counted 7 days after adding puromycin (**Figure 1E**). When the cells were induced with 3 factors (without c-Myc), the PαS cells yielded not only more ES-like colonies counted 42 days after retroviral transduction, but also more GFP^+^/DsRed^−^ colonies, compared to the OP cells, TTFs, or MEFs using 3 or 4 factors, counted 7 days after the addition of puromycin (**Figure 1F and G**). We also confirmed the higher induction efficiency of PαS cells compare to TTFs by using integration-free method (**Table S1**), and immune-deficient NOD/Shi-*scid* and IL-2Rγ^null^ (*NOG*) mice that is a well known non-permissive strain (Matsui et al. manuscript in preparation).

### Characterization of iPS clones derived from PαS, OP or TTF

**Figure 2:**
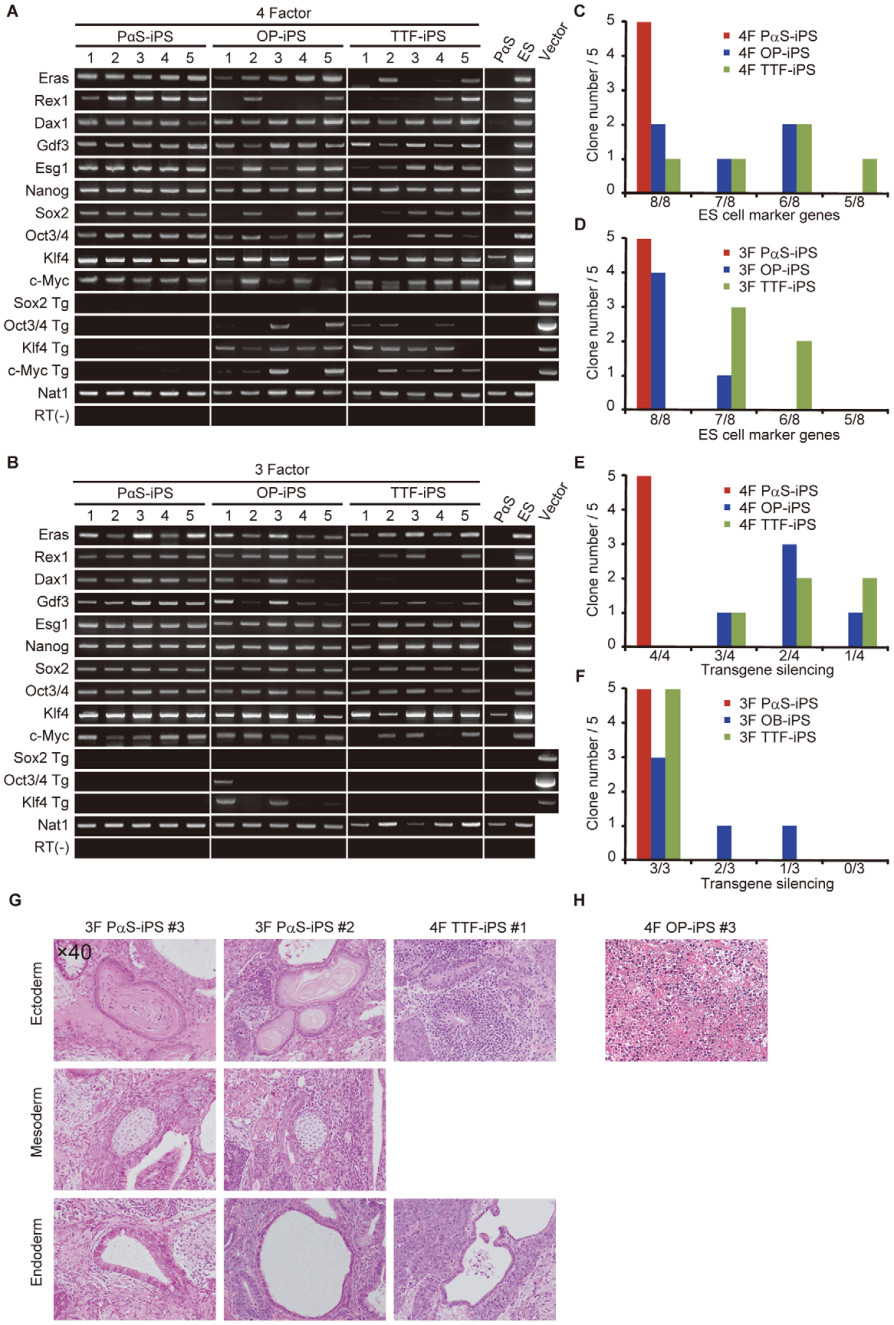
Gene expression and in vivo differentiation of iPS cells. RT-PCR of 4F-iPS cells (**A**) and 3F-iPS cells (**B**) for ES-cell marker genes, including Eras, Rex1, Dax1, Gdf3, Esg1, Nanog, Oct3/4, and Sox2 and to detect silencing of the transgenes. **C**, Number of ES-cell marker genes expressed using 4F induction. **D**, Number of ES-cell marker genes expressed using 3F induction. **E**, Transgenes silenced in 4F induction. **F,** Transgenes silenced in 3F induction. **G**, Teratomas from iPS cells, transplanted subcutaneously into nude mice. After 4-6 weeks, the teratomas were analyzed histologically with haematoxylin and eosin staining. TTF-iPS 4F1 did not differentiate mesoderm. **H**, Undifferentiated cells from 4F OP-iPS #3.

Next, we randomly selected five GFP^+^/DsRed^−^ clones from each adult cell source (PαS, OP, and TTF) and verified the quality of individual clones. We first analyzed the marker-gene expression for undifferentiated ES cells and transgene silencing by RT-PCR. All 4-factor (4F-) and 3-factor (3F-) induced P(S-iPS clones uniformly expressed all the ES-cell marker genes (Figure 2A–D), but completely lost all transgene expression (**Figure 2E and F**). In addition, although we chose DsRed^−^ clones, the transgene silencing was incomplete in several OP-iPS and TTF-iPS clones (**Figure 2E and F**). The PαS-iPS clones were also homogenously positive for the immature ES-cell markers alkaline phosphatase (ALP) and SSEA-1, but some of the OP-/TTF-iPS clones showed a heterogeneous phenotype for SSEA-1 expression (**Figure S2A and B**).

We next examined the pluripotency of each iPS clone by teratoma formation. All of the 4F-/3F-induced PαS-iPS clones formed teratomas, although 4F-OP-iPS #3 and 4F-TTF-iPS #1 clones did not differentiate into all three germ layers or remained undifferentiated cells. (**Figure 2G and H** **, Figure S3**). Interestingly, some of the OP-/TTF-iPS clones that lacked several ES-cell marker genes still differentiated into all three germ layers as teratomas (**Figure 2A–F**).

**Figure 3:**
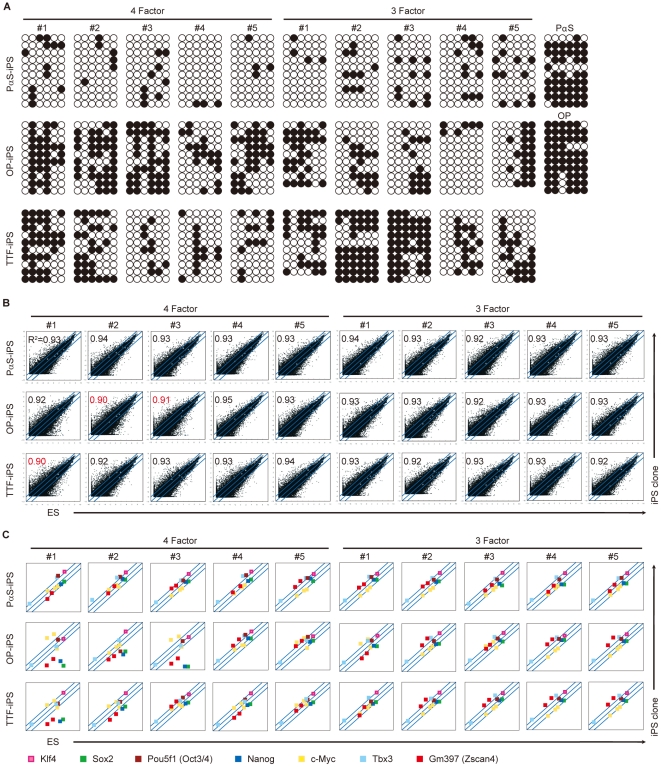
Characterization of iPS cells and PαS cells. **A**, DNA methylation of the promoter region of the Nanog gene. White circles indicate unmethylated CpG dinucleotides; black circles indicate methylated CpG dinucleotides. **B**, Scatter plots showing the comparison of global gene expression between ES cells and PαS-iPS, OP-iPS, and TTF-iPS cells determined by DNA microarray. Red number; R^2^ values over 0.92 compared with ES cells as positive. **C**, Microarray Scatter Plots were especially showing klf4, Sox2, Pou5f1 (Oct3/4), Nanog, c-Myc, Tbx3, and GM397 (Zscan4) gene.

Bisulfate genomic sequencing of the Nanog promoter revealed that the primary PαS and OP cells were similarly methylated as TTFs [20] and hepatocytes [4] (**Figure 3A**). DNA microarray analysis confirmed that all of the PαS-iPS clones had similar ES-cell-like gene expression profiles detected by R^2^ value (**Figure 3B**). From Microarray analysis, two of the OP and one of the TTF clones that expressed relatively low levels of Sox2, Nanog, and Zscan4 [21] also showed heavy methylation of the Nanog promoter legion. Also, two of these clones (4F-OP-iPS #3 and 4F-TTF-iPS # 1) did not show pluripotency in the teratoma assay. Therefore, the global gene expression profile and Nanog promoter methylation patterns correlated with the functional quality of these clones. However, two of the heavily methylated TTF-derived clones had ES-cell-like gene expression profiles, including Nanog (**Figure 3A and C**) and formed three-germ-layer teratomas (**Figure S3**). Collectively, our data showed that iPS clones generated from PαS cells had uniform profiles in all the assays, whereas the OP-/TTF-derived iPS clones showed divergence, even among those that were isolated by Nanog-puromycin resistance GFP^+^DsRed^−^ selection (**Table S2**).

### Chimeras from PαS-iPS cells

We next investigated whether the PαS-iPS cells were of a sufficient quality to produce adult chimeras. From previous examinations, OP-iPS clones and TTF iPS clones could not pass the total quality assessment, excluding 3F-OP-iPS #4 (**Table S1**). We selected three 4F-PαS-iPS, three 3F-PαS-iPS, and one from each 3F-OP-iPS and 4F-TTF-iPS **(Table S2**). These cells were introduced into eight-cell-stage ICR embryos by aggregation [22]. The P(S-iPS cells were well integrated into the inner cell mass during the maturation process, whereas a large number of OP-iPS and TTF-iPS cells were not (**Figure S4**). Two of the three 4F-PαS-iPS and all of the 3F-PαS-iPS contributed to chimeras. Most of the PαS-iPS clones competently generated adult chimeric mice, whereas none of the OP-/TTF-iPS-aggregated embryos resulted in chimeric mice.

**Figure 4:**
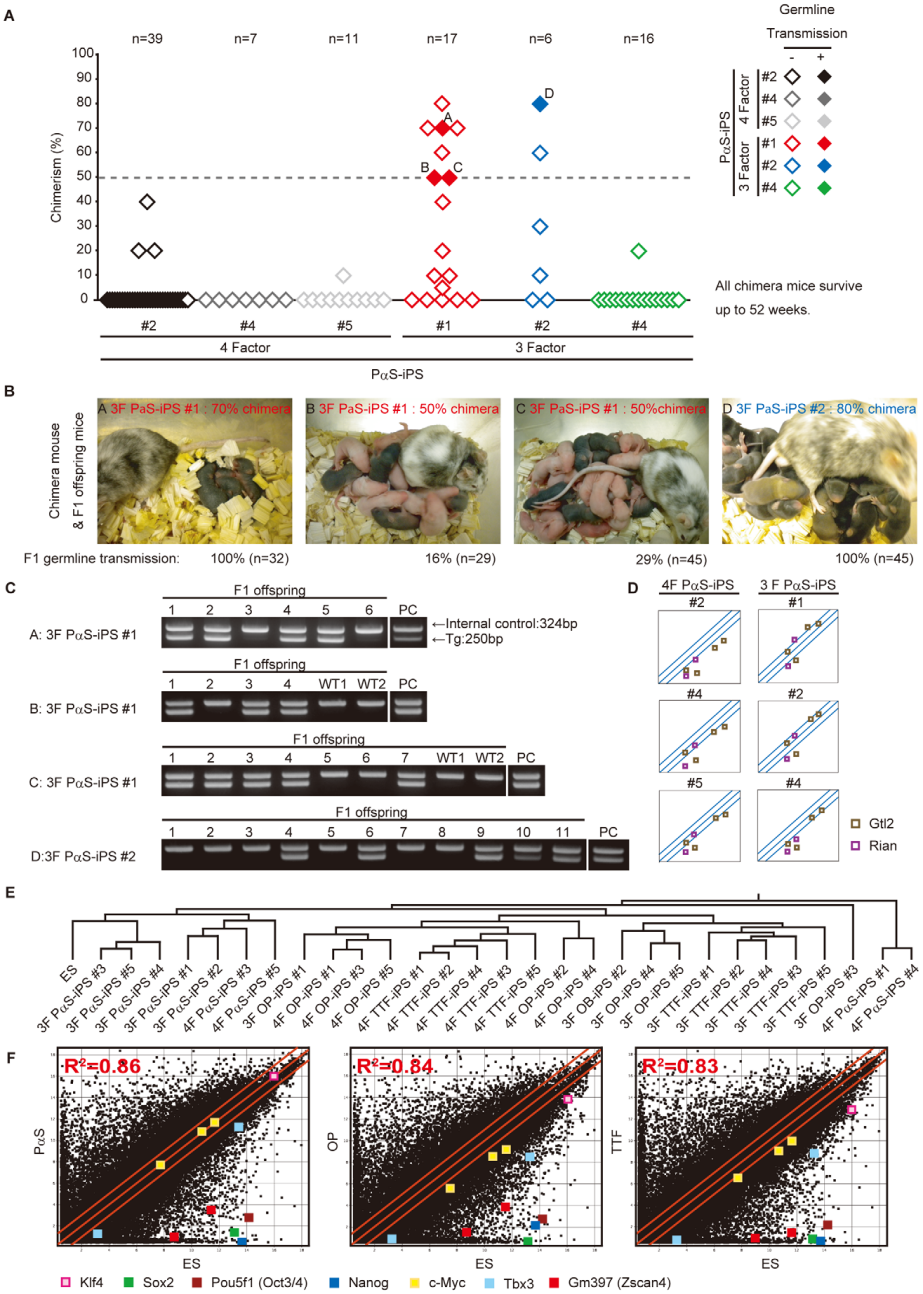
Germline chimeras and offspring from PαS-iPS cells. **A**, Chimeric and F1 mice from 4F-PαS-iPS and 3F-PαS-iPS cells. 3F-PαS-iPS could observe nine over 50% chimerism chimeras. **B**, Germline-competent chimeric mice and F1 mice. Seven chimeras (>50% coloured coat colour) from 3F-PαS-iPS #1 and two from 3F-PαS-iPS #2 were bred with ICR females, and three of seven chimeas from 3F-PαS-iPS #1 and one of two chimera from 3F-PαS-iPS #2 produced iPS derived F1 mice. The ratio of pigmented F1 mice was observed. Numbers below each panel give the percentage of pigmented F1 mice. **C**, PCR analysis showing the presence of the GFP cassette in F1 mice obtained from the intercross between a chimeric male and an ICR female. **D**, Scatter plots compared the Gtl2 and Rian gene expression between 4F/3F PαS-iPS and ES cells. **E**, Hierarchical clustering comparing the global gene expression between ES and iPS cells determined by DNA microarray. **F**, Scatter plots comparing the global gene expression between ES cells and source cells including PαS cells, OP cells, and TTF cells determined by DNA microarray.

Interestingly, the 3F-PαS-iPS clones clearly showed higher chimerism efficiency than the 4F-clones (**Figure 4A**). This suggested that the 3F-PαS-iPS cells were more efficient in colonizing germ tissues. Therefore, using one of the most stringent criteria for demonstrating the quality of iPS clones [23], we next tested the frequency of their germ-line transmission and the production of viable F1 offspring.

Five of the nine chimeric animals were infertile, which could be explained by hermaphroditism of the chimeric mice [24], since both of the iPS clones were of male origin. The other four chimeras produced litters of which two had 100% black offspring, and two had between 16% and 29% black offspring (**Figure 4B**).

Nanog-GFP-IRES-Puro^r^ transgene integration was identified by genomic DNA PCR in 60% of the black offspring. Since the original PαS cells were derived from heterozygous Tg animals, approximately half of the F1 offspring were expected to possess the integrated transgene (**Figure 4C**). In the germ-line competent 3F-PαS-iPS cells, the expression levels of Gtl2 and Rian, which are associated with germ-line contribution, were similar to those of ES cells, but their levels were slightly lower in #4 (**Figure 4D**).

The DNA microarray hierarchical clustering data confirmed that the 3F-PαS-iPS clones were the closest to ES cells among the clones derived from the various cell sources, and even the 4F-PαS-iPS cells (**Figure 4E**).

## Discussion

Here we demonstrated that highly enriched mesenchymal stem cells (PαS cells) produce high-quality iPS clones at a high frequency. All the PαS-derived clones we tested had immature ES-cell-like characteristics, according to their gene expression, *in vivo* pluripotency, and the competency to produce adult chimeric mice. In addition, we have not observed the early death of chimeric mice, as was previously reported [4], in either the 3F- or 4F-PαS-iPS-containing chimeras, which have lived more than 52 weeks, to date. This is another advantage of using purified MSCs as the iPS cell source. Selection markers driven by the Nanog/Oct4 promoter allowed us to obtain high quality iPS cells at a higher frequency than Fbx15 promoter selection [1], [19]. However, it is well known that most Nanog/Oct4-GFP-positive clones are not in a fully reprogrammed state, and are therefore termed “junk” cells [25]. Therefore, a series of screening processes is required to ensure the iPS cells are “qualified.” Using purified MSCs as a cell source may relieve the researcher of this tedious process.

Our results also revealed that 3F induction (without c-Myc) was more efficient for the germ-line transmission of PαS-iPS. This was an interesting observation, since it is known that 3F-induced MEFs or TTFs rarely produce germ-line transmissible iPS clones [25], [26]. As already reported, mouse and human NSCs can be reprogrammed with Oct4 and Klf4 only, since NSCs intrinsically express Sox2 and c-Myc [7], [8]. Our DNA microarray analysis revealed that the original PαS cells expressed c-Myc, Klf4, and Tbx3 [26] mRNA at similar levels as ES cells, but their levels in the OP cells and TTFs were lower (**Figure 4F**). This was also consistent with our observation that c-Myc transduction was not required for the complete reprogramming of PαS cells. A recent publication demonstrated that germ-line-competent iPS cells are easily obtained when c-Myc is replaced by Tbx3 [26]. That PαS cells and ES cells express equivalent levels of both c-Myc and Tbx3 may be another reason for the enhanced reprogramming and germ-line transmission of the PαS cells. However, unlike with NSCs, we could not obtain iPS cells from PαS cells when Klf4 or c-Myc and Klf4 were not among the transduced factors. These results suggest that exogenous c-Myc is dispensable, but Klf4 is required for deriving iPS cells from PαS cells.

We conclude that immature tissue stem cells (PαS cells) are a more efficient source of iPS cells than partially committed ones (OP cells) or a mixed cell population (TTFs) of the same lineage. Among the various tissue stem cells that can be isolated from adults, MSCs in the bone marrow are the most easily accessible, and can be obtained less invasively than NSCs or stomach cells[4], [7], [8], [9]. If human MSCs show the same features as murine MSCs, the clinical implications of this finding will be immense, not only because human MSCs would be an ideal source of high-quality iPS cells, but also because existing bone-marrow banks could be exploited to prepare HLA-specific iPS cells for use in regenerative medicine.

## Materials and Methods

### Preparation of bone-marrow cell suspension

The mice were kept under specific pathogen-free conditions in our animal facility at keio University School of Medicine. All experimental procedures and protocols were approved by the ethics committee of Keio University and were in accordance with the Guide for the Care and Use of Laboratory Animals. Approval ID: 09089-(8). Mouse femurs and tibias were dissected out and crushed with a pestle. The crushed bones were washed in HBSS^+^ (Gibco) supplemented with 2% FBS, 10 mM HEPES, and 1% Penicillin/Streptomycin(P/S) to remove the hematopoietic cells. The bone fragments were incubated for 1 hr at 37°C in 0.2% collagenase (Wako) in DMEM (Gibco) containing 10 mM HEPES and 1% P/S. The suspension was filtered through a cell strainer (Falcon) and collected by centrifugation at 280×g for 7 min at 4°C. The pellet was resuspended for 5–10 s in 1 ml water to burst red blood cells, after which 1 ml of 2 × PBS containing 4% FBS was added. The cells were resuspended in HBSS^+^ and poured through a cell strainer.

### Flow cytometry analysis and cell sorting

The following fluorescently conjugated antibodies (PE, APC, or FITC) were used for analysis and cell sorting: PE-conjugated CD45 (30-F11), TER119 (TER-119), and SSEA-1, APC-conjugated PDGFRα (APA5), and FITC-conjugated Sca-1 (Ly6A/E). Flow-cytometry analysis and sorting were performed on a triple-laser MoFlo (Dako) or JSAN (Bay Bioscience) flow cytometer. PI fluorescence was measured, and a live cell gate was defined that excluded the cells positive for PI. Additional gates were defined as positive for PDGFRα and Sca-1 and negative for CD45 and TER119, according to the isotype control fluorescence intensity. Live iPS cells gated in the PI-negative region were stained for SSEA-1 for analysis.

### Cell culture

Mouse MSCs were maintained in MEM-α containing 10%FBS, 1% P/S, and 10 mM HEPES. The iPS cells were maintained in ES medium (DMEM containing 15% FBS, 1 × NEAA, 1 mM sodium pyruvate, 5.5 mM 2-ME, 50 units ml^−1^ penicillin, and 50 µgml^−1^ streptomycin) on feeder layers of mitomycin-C-treated SNL cells into which we had stably incorporated the puromycin-resistance gene. As a source of LIF, we used conditioned medium from Plat-E cell cultures that had been transduced with a LIF-expressing vector. Plat-E cells, which were also used to produce retroviruses, were maintained in DMEM containing 10% FBS, 50 unitsml^−1^ penicillin, 50 µgml^-1^ streptomycin, 1 µgml^−1^ puromycin, and 10 µgml^−1^ blasticidin S. To establish TTFs, the tails from adult mice were peeled, minced into 1-cm pieces, placed on culture dishes, and incubated in MF-start medium (Toyobo) for 5 days. Cells that migrated out of the tail pieces were transferred to new plates (Passage 2) and maintained in DMEM containing 10% FBS. TTFs at Passage 3 were used to make iPS cells. ES and iPS cells were cultured as previously described[1].

### Generation of induced pluripotent stem cells

The iPS induction was performed as described previously[1], [15] with some modifications. Briefly, PαS cells, OP cells, TTFs, and MEFs were isolated from 6-week-old Nanog-reporter mice. Plat-E cells were seeded at 8×10^6^ cells per 100-mm dish. On the next day, 9 µg of pMX-based retroviral vectors for DsRed, Oct3/4, Sox2, Klf4, and c-Myc were individually introduced into separate dishes of Plat-E cells using 27 µl of FuGENE 6 transfection reagent. After 24 h, the medium was replaced with 10 ml of DMEM containing 10% FCS. The source cells were seeded at 1×10^4^ cells per 60-mm dish covered with feeder cells. On the next day, virus-containing supernatants from the Plat-E cultures were recovered and filtered through a 0.45-µm cellulose acetate filter. Equal volumes of the supernatants were mixed and supplemented with polybrene at a final concentration of 4 µgml^−1^. We prepared the PαS cells, OP cells, and TTFs concurrently. Two weeks after the cell expansion following cell sorting, we transduced the three types of source cells with 4 or 3 factors (without c-Myc) and DsRed as a marker for infection efficiency and transgene silencing. The source cells were incubated in the virus/polybrene-containing supernatants for 24 h. Two days after infection, the medium was changed to ES medium supplemented with LIF. Three days after infection, 1×10^4^ DsRed^+^-infected cells were plated on feeder cells. Three weeks after infection, the 4F cells were selected by puromycin (Sigma); four weeks after infection, the 3F cells were selected. One week after puromycin selection, the total colony number was counted. For Nanog iPS cells, puromycin (Sigma) was added at a final concentration of 1.5 µgml^−1^.

### RT-PCR

Total RNA was purified with Trizol (Invitrogen) and treated with a Turbo DNA-free kit (Ambion) to remove genomic DNA contamination. The total RNA was used for a transcription reaction with random primers (Stratagene), according to the manufacturer's instructions. PCR was performed with ExTaq (Takara, Japan).

### Teratoma formation and histological analysis

ES cells or iPS cells were suspended at 1×10^7^ cells/ml in DMEM containing 10% FBS. Nude mice were anesthetized with diethyl ether. Then, 100 µl of the cell suspension (1×10^6^ cells) was injected subcutaneously into the dorsal flank. Four weeks after the injection, the tumours were surgically dissected from the mice, weighed, fixed in PBS containing 4% formaldehyde, embedded in paraffin, and sectioned. The sections were stained with haematoxylin and eosin. Injection was done three times for all clones.

### Bisulfite genomic sequencing

Bisulfite treatment was performed using the CpGenome modification kit (Chemicon) according to the manufacturer's recommendations. The treated DNA was purified with a QIAquick column (Qiagen). The amplified products were cloned into pCR2.1-TOPO (Invitrogen). Ten randomly selected clones were sequenced with the M13 forward and M13 reverse primers for each gene.

### DNA microarray

The total RNA from ES cells, PαS cells, OP cells, TTFs, and iPS cells was labelled with Cy3. Samples were hybridized to a Mouse Oligo Microarray (Agilent) according to the manufacturer's protocol. The arrays were scanned with a G2565BA Microarray Scanner System (Agilent). Data were analysed using GeneSpring GX software (Agilent). Genes for which the value fluctuated more than twofold between duplicated analyses were excluded. For hierarchical clustering, we use Pearson's correlation for similarity measure and for average linkage clustering. GEO accession number: GSE23717.

### Chimera formation

iPS cells were aggregated with zona-pellucida-free eight-cell-stage embryos to generate chimeras. First, two-cell embryos were flushed from ICR females at 1.5 dpc and cultured in microdrops of mWitten medium until the eight-cell stage. The zona pellucida was dissolved in Acid Tyrode's solution (Sigma). After a short treatment with trypsin (Nacalai Tesque), the iPS cells were transferred into the microdrops of mWitten medium to make contact with the denuded eight-cell embryos. Eight-cell embryos aggregated with iPS cells were cultured overnight at 37°C, 5% CO_2_. The aggregated morulae or blastocysts were transferred into the oviducts of 0.5 dpc pseudopregnant females.

## Supporting Information

Figure S1 **Infection efficiency of retrovirus.** DsRed expression was observed in transduced source cells. Infection efficiency was 81.5% of PαS cells, 85.3% of OP cells, and 80.7% of TTF cells. Left: PαS cells. Middle: OP cells. Right: TTF cells. Top: Phase contrast. Bottom: DsRed fluorescence. Bar, 250 µm.
(TIF)

Figure S2 **Characterization of iPS cell lines. A**, Expression of the pluripotency-associated gene alkaline phosphatase in iPS cells derived from TTF, OP, and PαS cells. Top: PαS-iPS clones. Middle: OP-iPS clones. Bottom: TTF-iPS clones. Bar, 200 µm. **B**, Histogram showed flow-cytometry analysis of the typical ES-cell surface antigen SSEA-1. Top: PαS-iPS clones. Middle: OP-iPS clones. Bottom: TTF-iPS clones. Blue line: experimental control. Red line: sample stained SSEA-1.
(TIF)

Figure S3 **Teratoma formation from iPS cells.** iPS cells were subcutaneously transplanted into nude mice. After 4–6 weeks, the teratomas were analyzed histologically with haematoxylin and eosin staining. 4F-OP-iPS #3 did not show differentiation potential for three germ layers. 4F-TTF-iPS #1 did not differentiate to mesoderm. Top: PαS-iPS clones. Middle: OP-iPS clones. Bottom: TTF-iPS clones. Upper: Ectoderm. Center: Mesoderm. Lower: Endoderm.
(TIF)

Figure S4 **Eight-cell-stage aggregation.** The iPS clones were transferred into eight-cell-stage ICR embryos by aggregation and cultured *in vitro* to blastocysts. Left: 3F-PαS-iPS #1. Middle: 3F-OP-iPS #4. Right: 3F-TTF-iPS #5. Top: Phase contrast. Middle: EGFP expression. Bottom: Merged image.
(TIF)

Table S1 **Induction efficiency of infected SeV infection.** PαS cells and TTF cells were infected with Sendai virus (SeV) [27]. Multiplicity of infection (MOI) was changed to test optimum density for generating iPS cells. Experiment 1 (Exp.01): Cells were seeded on 12 well plates (0.99×10^4^ cells/well). Experiment 2 (Exp.02): PαS cells were seeded on 6 cm dishes (5.0×10^4^ cells/well). TTF cells were seeded on 6 well plates (2.0×10^4^ cells/well). Experiment 3 (Exp. 03): Cells were seeded on 12 well plates (0.82×10^4^ cells/well). * cell aggregation. ** c-Myc was infected with MOI = 2.79 *** c-Myc was infected with MOI = 4.65.
(TIF)

Table S2 **Results summary of quality assessment.** All clones expressed alkaline phosphatase. SSEA-1 expression was detected in over 1% of positive cells compared with control cells. ES marker gene expression was detected by RT-PCR. Transgene silencing was detected by RT-PCR. Teratoma formation was detected by three times injection. Two clones could not differentiate into three germ-layers. Count for demethylation of Nanog promoter was over 80%. From microarray analysis, although all clones were over 0.9 R^2^ value, we considered R^2^ values over 0.92 compared with ES cells as positive. Chimera mouse was detected by court colour. Germ-line transmission was detected by court colour of offspring to mating over 50% chimerism Chimera mice with Wild type mice. ND: Not Done.
(TIF)

## References

[1] Takahashi K, Yamanaka S (2006). Induction of pluripotent stem cells from mouse embryonic and adult fibroblast cultures by defined factors.. Cell.

[2] Takahashi K, Tanabe K, Ohnuki M, Narita M, Ichisaka T (2007). Induction of pluripotent stem cells from adult human fibroblasts by defined factors.. Cell.

[3] Nakagawa M, Koyanagi M, Tanabe K, Takahashi K, Ichisaka T (2008). Generation of induced pluripotent stem cells without Myc from mouse and human fibroblasts.. Nat Biotechnol.

[4] Aoi T, Yae K, Nakagawa M, Ichisaka T, Okita K (2008). Generation of pluripotent stem cells from adult mouse liver and stomach cells.. Science.

[5] Stadtfeld M, Brennand K, Hochedlinger K (2008). Reprogramming of pancreatic beta cells into induced pluripotent stem cells.. Curr Biol.

[6] Hanna J, Markoulaki S, Schorderet P, Carey BW, Beard C (2008). Direct reprogramming of terminally differentiated mature B lymphocytes to pluripotency.. Cell.

[7] Kim JB, Sebastiano V, Wu G, Arauzo-Bravo MJ, Sasse P (2009). Oct4-induced pluripotency in adult neural stem cells.. Cell.

[8] Kim JB, Zaehres H, Wu G, Gentile L, Ko K (2008). Pluripotent stem cells induced from adult neural stem cells by reprogramming with two factors.. Nature.

[9] Miura K, Okada Y, Aoi T, Okada A, Takahashi K (2009). Variation in the safety of induced pluripotent stem cell lines.. Nat Biotechnol.

[10] Pittenger MF, Mackay AM, Beck SC, Jaiswal RK, Douglas R (1999). Multilineage potential of adult human mesenchymal stem cells.. Science.

[11] Prockop DJ (1997). Marrow stromal cells as stem cells for nonhematopoietic tissues.. Science.

[12] Friedenstein AJ, Deriglasova UF, Kulagina NN, Panasuk AF, Rudakowa SF (1974). Precursors for fibroblasts in different populations of hematopoietic cells as detected by the in vitro colony assay method.. Exp Hematol.

[13] Morikawa S, Mabuchi Y, Niibe K, Suzuki S, Nagoshi N (2009). Development of mesenchymal stem cells partially originate from the neural crest.. Biochem Biophys Res Commun.

[14] Morikawa S, Mabuchi Y, Kubota Y, Nagai Y, Niibe K (2009). Prospective identification, isolation, and systemic transplantation of multipotent mesenchymal stem cells in murine bone marrow.. J Exp Med.

[15] Takahashi K, Okita K, Nakagawa M, Yamanaka S (2007). Induction of pluripotent stem cells from fibroblast cultures.. Nat Protoc.

[16] Cherry SR, Biniszkiewicz D, van Parijs L, Baltimore D, Jaenisch R (2000). Retroviral expression in embryonic stem cells and hematopoietic stem cells.. Mol Cell Biol.

[17] Chambers I, Colby D, Robertson M, Nichols J, Lee S (2003). Functional expression cloning of Nanog, a pluripotency sustaining factor in embryonic stem cells.. Cell.

[18] Mitsui K, Tokuzawa Y, Itoh H, Segawa K, Murakami M (2003). The homeoprotein Nanog is required for maintenance of pluripotency in mouse epiblast and ES cells.. Cell.

[19] Okita K, Ichisaka T, Yamanaka S (2007). Generation of germline-competent induced pluripotent stem cells.. Nature.

[20] Stadtfeld M, Nagaya M, Utikal J, Weir G, Hochedlinger K (2008). Induced pluripotent stem cells generated without viral integration.. Science.

[21] Zalzman M, Falco G, Sharova LV, Nishiyama A, Thomas M (2010). Zscan4 regulates telomere elongation and genomic stability in ES cells.. Nature.

[22] Wood SA, Allen ND, Rossant J, Auerbach A, Nagy A (1993). Non-injection methods for the production of embryonic stem cell-embryo chimaeras.. Nature.

[23] Daley GQ, Lensch MW, Jaenisch R, Meissner A, Plath K (2009). Broader implications of defining standards for the pluripotency of iPSCs.. Cell Stem Cell.

[24] Tarkowski AK (1998). Mouse chimaeras revisited: recollections and reflections.. Int J Dev Biol.

[25] Stadtfeld M, Apostolou E, Akutsu H, Fukuda A, Follett P (2010). Aberrant silencing of imprinted genes on chromosome 12qF1 in mouse induced pluripotent stem cells.. Nature.

[26] Han J, Yuan P, Yang H, Zhang J, Soh BS (2010). Tbx3 improves the germ-line competency of induced pluripotent stem cells.. Nature.

[27] Fusaki N, Ban H, Nishiyama A, Saeki K, Hasegawa M (2009). Efficient induction of transgene-free human pluripotent stem cells using a vector based on Sendai virus, an RNA virus that does not integrate into the host genome.. Proc Jpn Acad Ser B Phys Biol Sci.

