# Supplementary figures and images for: Purified Mesenchymal Stem Cells Are an Efficient Source for iPS Cell Induction
Source: PLoS One. 2011 Mar 11;6(3):e17610. doi: 10.1371/journal.pone.0017610 (PMC3055883; doi:10.1371/journal.pone.0017610)

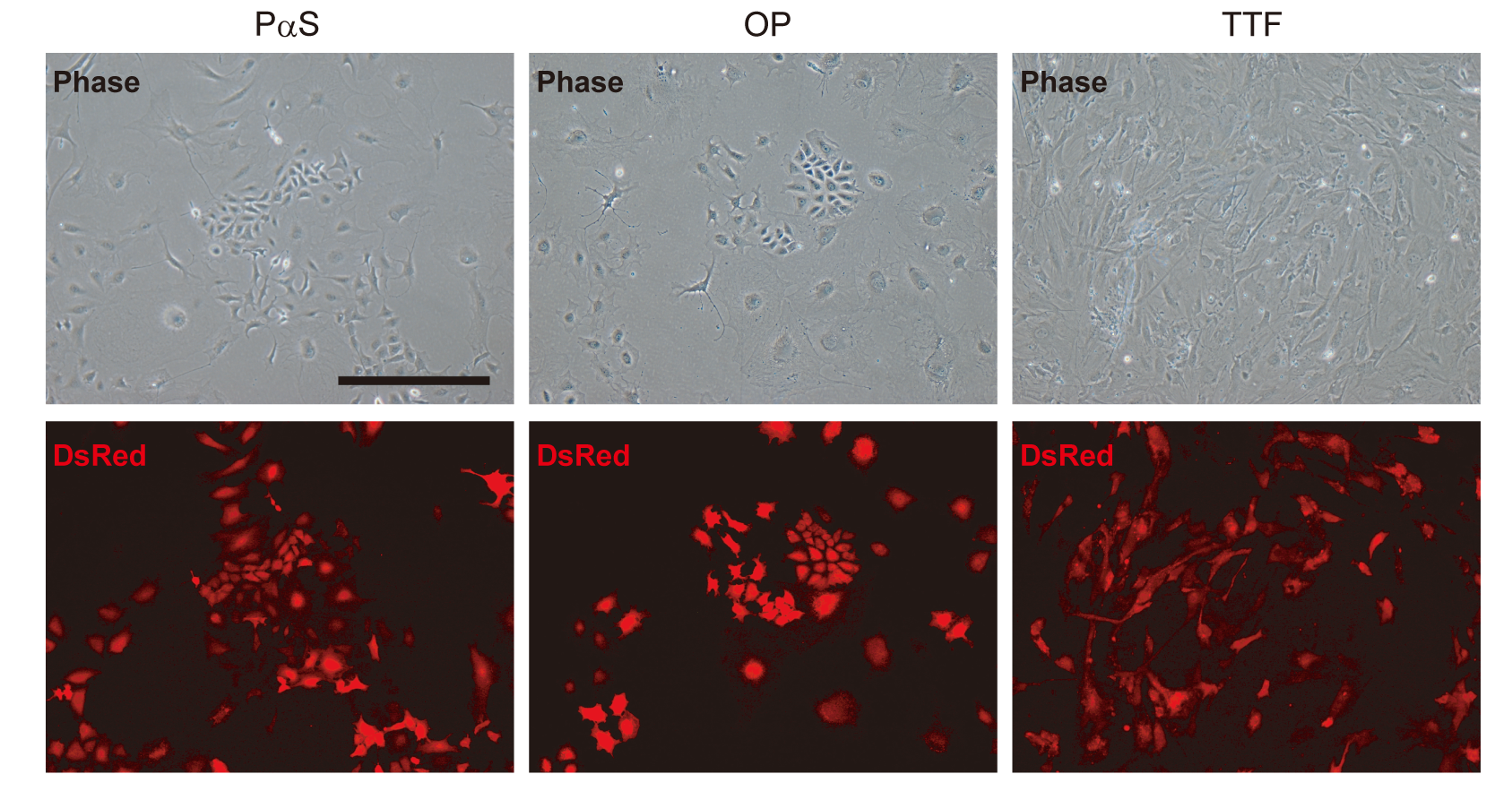

Supplement: Figure S1 — Infection efficiency of retrovirus. DsRed expression was observed in transduced source cells. Infection efficiency was 81.5% of PαS cells, 85.3% of OP cells, and 80.7% of TTF cells. Left: PαS cells. Middle: OP cells. Right: TTF cells. Top: Phase contrast. Bottom: DsRed fluorescence. Bar, 250 µm. (TIF) [file pone.0017610.s001.tif]

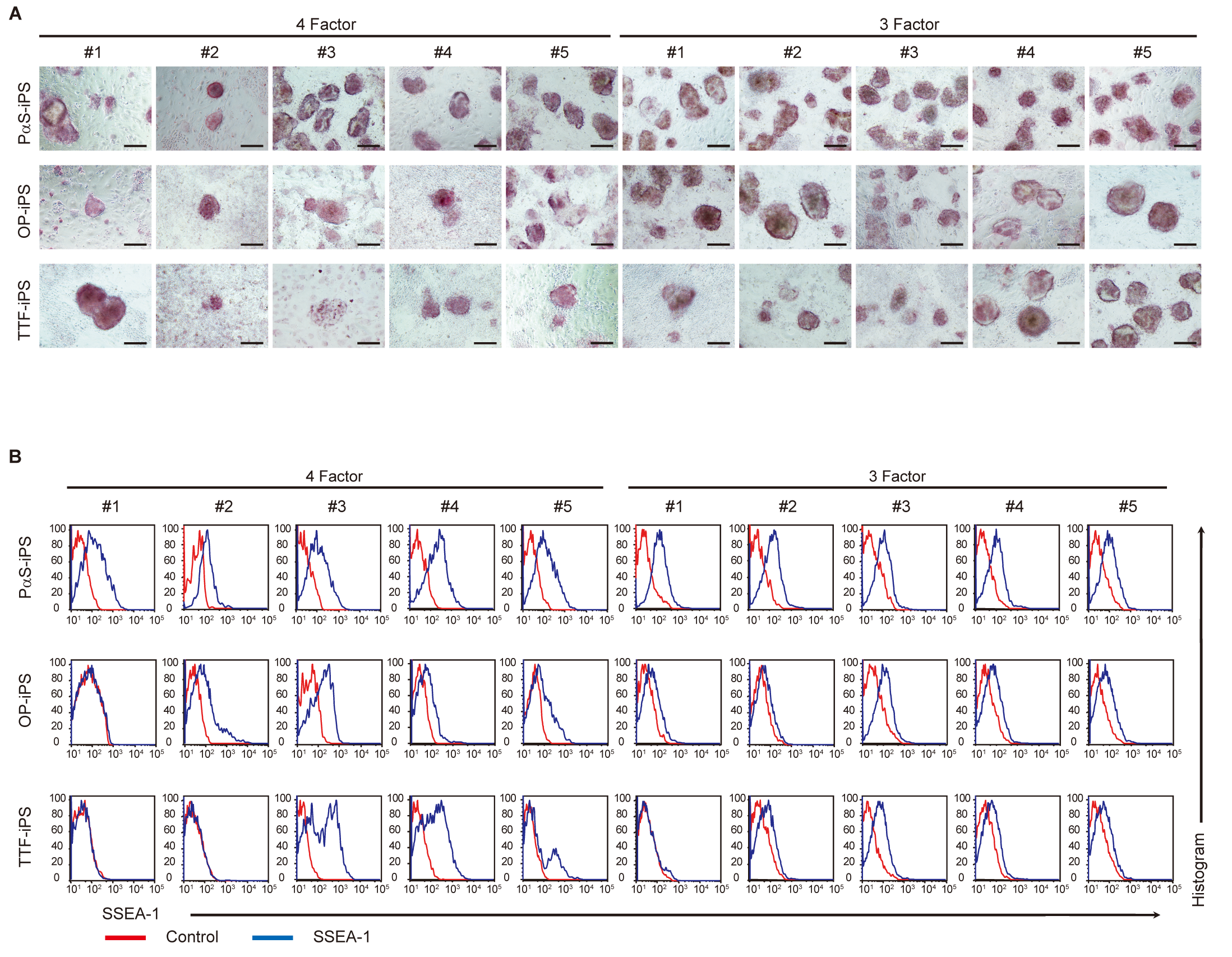

Supplement: Figure S2 — Characterization of iPS cell lines. A, Expression of the pluripotency-associated gene alkaline phosphatase in iPS cells derived from TTF, OP, and PαS cells. Top: PαS-iPS clones. Middle: OP-iPS clones. Bottom: TTF-iPS clones. Bar, 200 µm. B, Histogram showed flow-cytometry analysis of the typical ES-cell surface antigen SSEA-1. Top: PαS-iPS clones. Middle: OP-iPS clones. Bottom: TTF-iPS clones. Blue line: experimental control. Red line: sample stained SSEA-1. (TIF) [file pone.0017610.s002.tif]

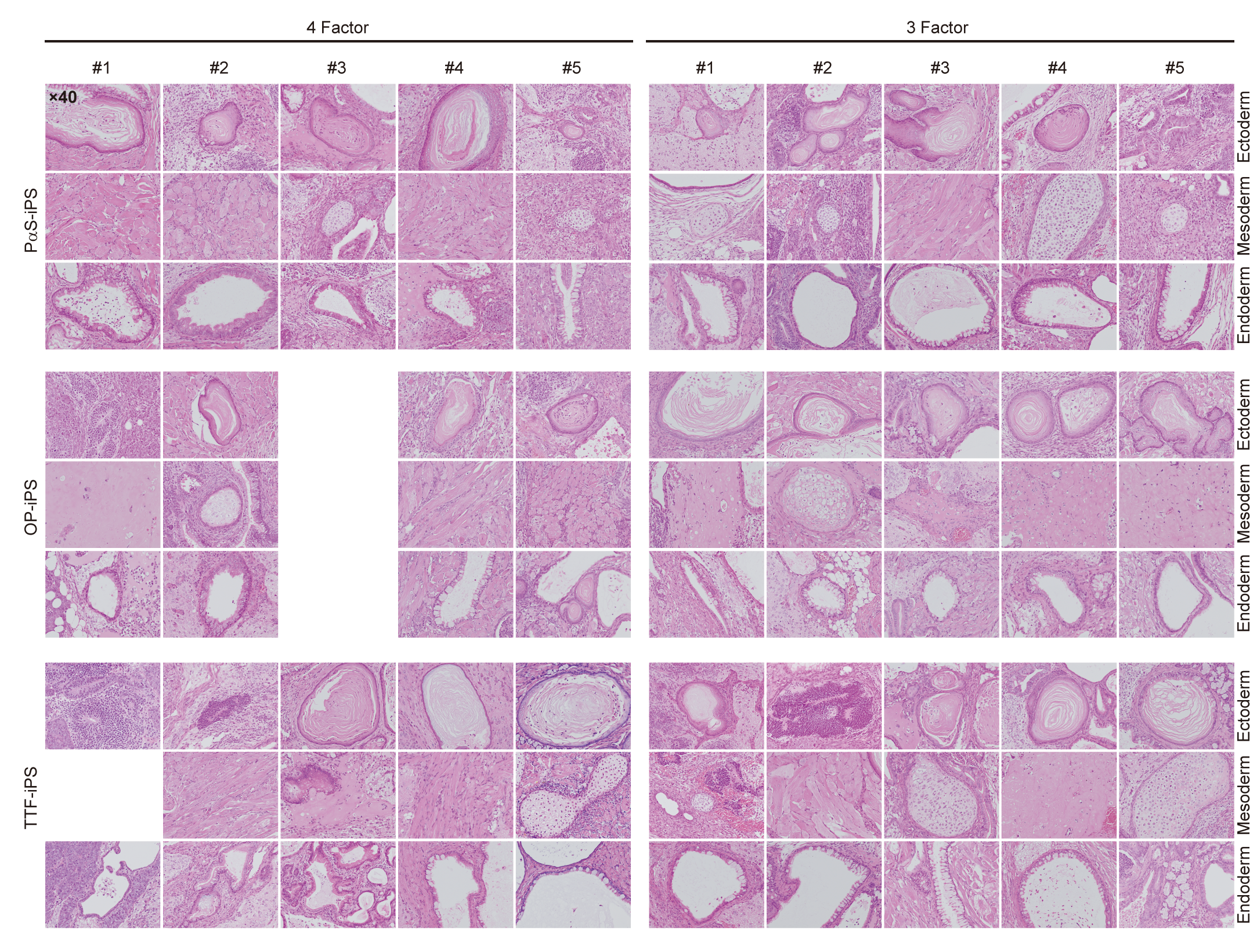

Supplement: Figure S3 — Teratoma formation from iPS cells. iPS cells were subcutaneously transplanted into nude mice. After 4–6 weeks, the teratomas were analyzed histologically with haematoxylin and eosin staining. 4F-OP-iPS #3 did not show differentiation potential for three germ layers. 4F-TTF-iPS #1 did not differentiate to mesoderm. Top: PαS-iPS clones. Middle: OP-iPS clones. Bottom: TTF-iPS clones. Upper: Ectoderm. Center: Mesoderm. Lower: Endoderm. (TIF) [file pone.0017610.s003.tif]

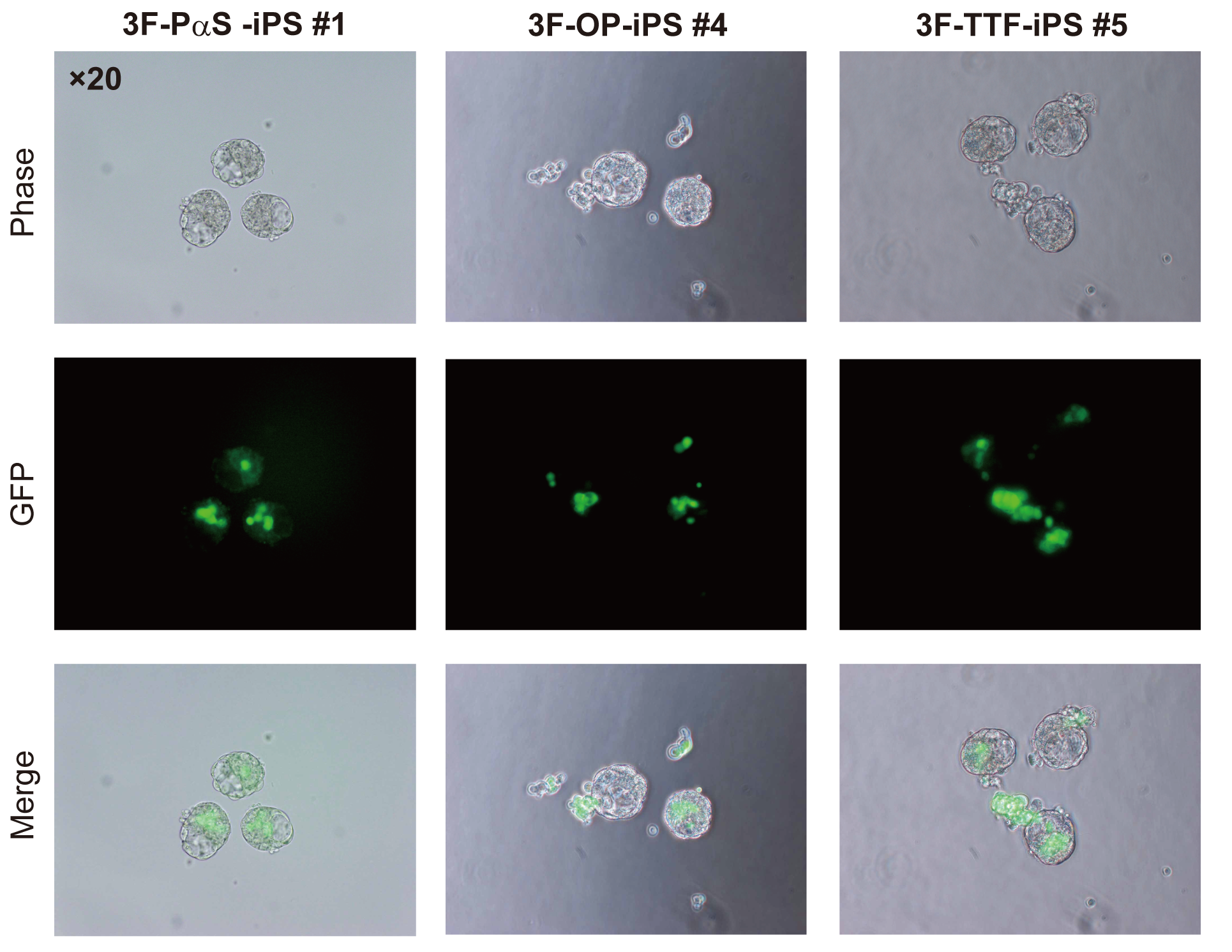

Supplement: Figure S4 — Eight-cell-stage aggregation. The iPS clones were transferred into eight-cell-stage ICR embryos by aggregation and cultured in vitro to blastocysts. Left: 3F-PαS-iPS #1. Middle: 3F-OP-iPS #4. Right: 3F-TTF-iPS #5. Top: Phase contrast. Middle: EGFP expression. Bottom: Merged image. (TIF) [file pone.0017610.s004.tif]

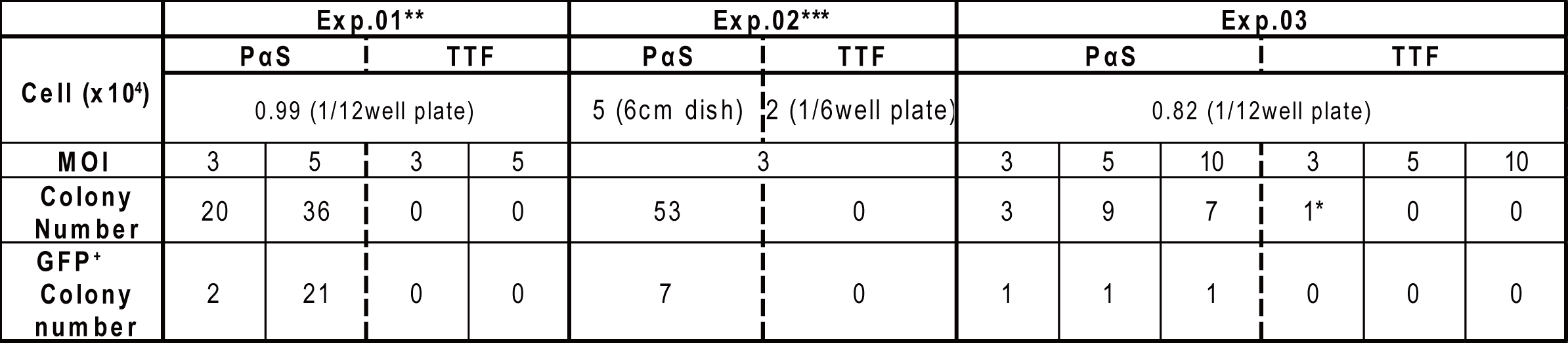

Supplement: Table S1 — Induction efficiency of infected SeV infection. PαS cells and TTF cells were infected with Sendai virus (SeV) [27]. Multiplicity of infection (MOI) was changed to test optimum density for generating iPS cells. Experiment 1 (Exp.01): Cells were seeded on 12 well plates (0.99×10^4^ cells/well). Experiment 2 (Exp.02): PαS cells were seeded on 6 cm dishes (5.0×10^4^ cells/well). TTF cells were seeded on 6 well plates (2.0×10^4^ cells/well). Experiment 3 (Exp. 03): Cells were seeded on 12 well plates (0.82×10^4^ cells/well). * cell aggregation. ** c-Myc was infected with MOI = 2.79 *** c-Myc was infected with MOI = 4.65. (TIF) [file pone.0017610.s005.tif]

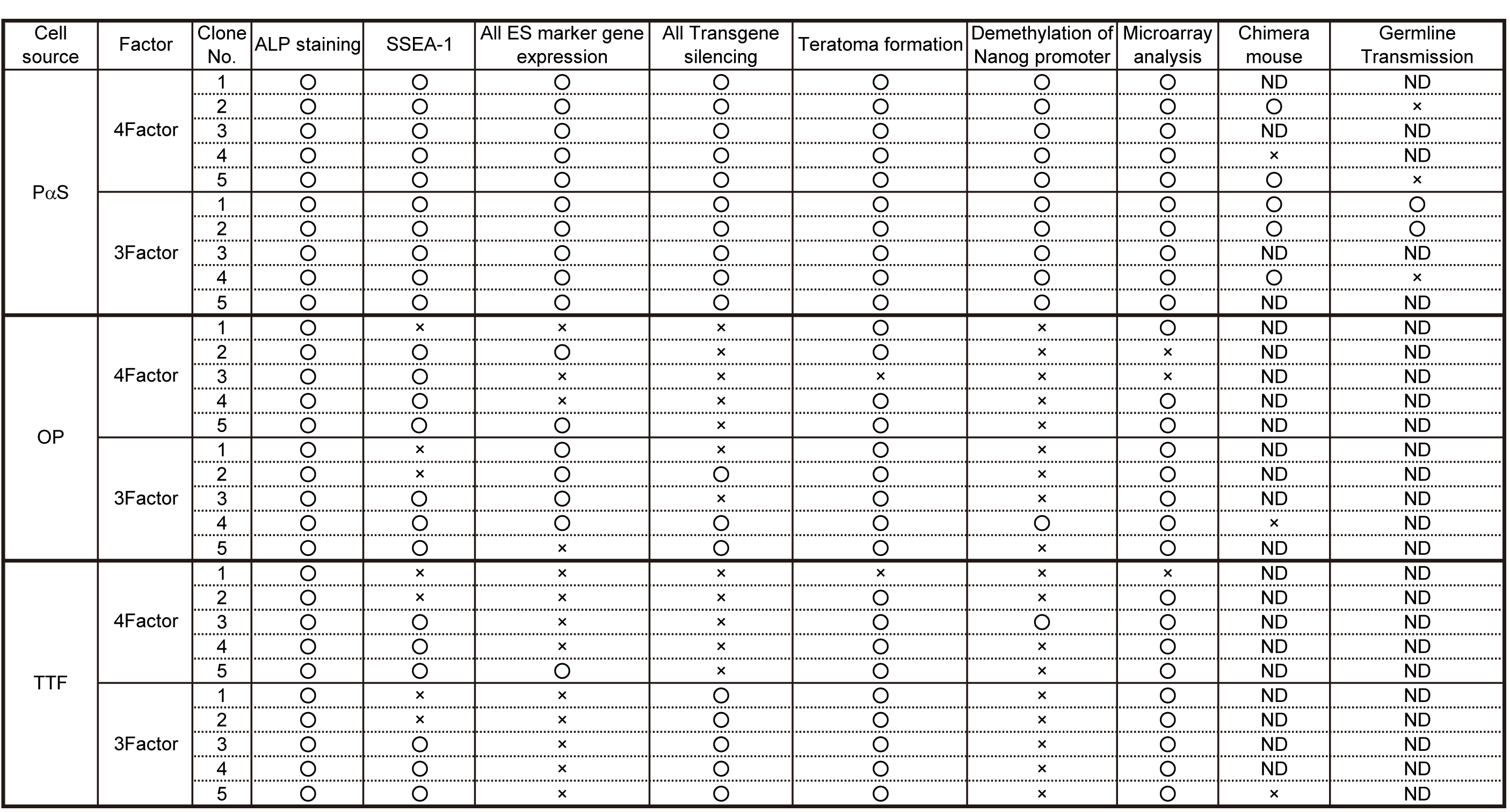

Supplement: Table S2 — Results summary of quality assessment. All clones expressed alkaline phosphatase. SSEA-1 expression was detected in over 1% of positive cells compared with control cells. ES marker gene expression was detected by RT-PCR. Transgene silencing was detected by RT-PCR. Teratoma formation was detected by three times injection. Two clones could not differentiate into three germ-layers. Count for demethylation of Nanog promoter was over 80%. From microarray analysis, although all clones were over 0.9 R2 value, we considered R2 values over 0.92 compared with ES cells as positive. Chimera mouse was detected by court colour. Germ-line transmission was detected by court colour of offspring to mating over 50% chimerism Chimera mice with Wild type mice. ND: Not Done. (TIF) [file pone.0017610.s006.tif]
